# Characterization of a novel unliked 12 X-STR typing assay for forensic purposes in an admixed Rio de Janeiro population sample

**DOI:** 10.1590/1678-4685-GMB-2025-0015

**Published:** 2025-12-12

**Authors:** Isadora C. de Toledo e Mello, Maria Clara da Costa Simas, Rosane Silva, Rodrigo Soares de Moura

**Affiliations:** 1 Universidade Federal do Rio de Janeiro, Instituto de Biofísica Carlos Chagas Filho, Rio de Janeiro, RJ, Brazil; 2 Universidade Federal do Rio de Janeiro, Instituto de Biologia, Rio de Janeiro, RJ, Brazil

**Keywords:** Massively Parallel Sequencing (MPS), capillary gel electrophoresis, isoalleles, X-STR markers, forensic

## Abstract

X-STR analysis offers novel pathways for exploring both paternal and maternal lineages, which is particularly valuable in parenthood testing where standard methods fail. Despite being limited in availability, these markers show promise in improving forensic analyses, especially with emerging technology such as Massively Parallel Sequencing (MPS). This study introduces a new X-STRs multiplex system that could increase discrimination power in complex cases, expanding marker diversity and complementing traditional markers. We selected 12 X-STR loci (DXS61071, DXS97199, DXS12310, DXS14221, DXS33963, DXS39152, DXS44734, DXS54471, DXS68748, DXS70370, DXS13932, and DXS14986) for the assay. Genomic DNA was extracted from 100 unrelated males and 104 females, in a sample population from Rio de Janeiro, amplified with custom-designed primers and subjected to capillary gel electrophoresis and MPS. Forensic efficiency evaluation revealed high values of the combined power of discrimination in males (cPDM ≥ 0.999999997) and females (cPDF ≥ 0.999999999999995), as well as combined paternity exclusion (cPE ≥ 0.99999). Among these markers, two exhibit high polymorphism, with multiple isoalleles distinguishable by their sequences, using MPS technology. The incorporation of these new X-STR markers multiplex system in conjunction with MPS analysis could potentially increase the power of discrimination in forensic analysis for mixtures and complex cases.

## Introduction

In forensic genetics, X-chromosomal Short Tandem Repeats (X-STRs) can provide valuable additional information, especially in complex kinship cases, thereby contributing significantly to resolving ambiguities and offering closure to individuals and families. STR markers have long been the main tool used by most laboratories to perform human identification and kinship analyses. However, in certain cases, autosomal STRs may yield inconclusive results. In such scenarios, it becomes essential to consider complementary genetic markers. X-STR markers were discovered along with the rise of PCR technology, sparking interest because of the various diseases and traits linked to the X-chromosome (Szibor, 2007). The distinctive inheritance patterns exhibited by X-chromosomes offer novel pathways for investigating both paternal and maternal lineages, an alternative to conventional lineage markers like Y-chromosomal haplotypes and mitochondrial (Tillmar et al., 2017; Gomes et al., 2020; Zhang et al., 2021). X-STR markers offer a notable advantage in parenthood testing by resolving cases where standard testing yields inconclusive or weak statistical results. The analysis of X‐STRs can strongly benefit the investigation of some complex kinship cases, for example in paternity tests for half-sisters when the alleged father’s DNA is not available. Also, when the putative father of a daughter cannot be tested (deficiency paternity cases), the daughter’s X-chromosome profile is compared to potential paternal relatives, such as the alleged father’s brothers or mother, to determine her paternal lineage (Edelmann et al., 2004; Edelmann *et al*., 2012; Abdulazeez et al., 2020). In some paternity cases, if the child shows a high rate of homozygosity on autosomal markers, it may raise concerns about an incestuous relationship (Woods et al., 2006) which involves first-degree relatives (father-daughter, mother-son, or brother-sister), who share 30-50% of their genes (Jakovski et al., 2011). In such cases, examining the daughter’s X-STR profile offers valuable insights. Also, in cases of father-daughter incest where the father is also the grandfather, the child either shares the mother’s genetic makeup or shows homozygosity for all X STR markers (Woods *et al*., 2006). When close blood relatives like father and son are potential fathers of the same child, the exclusion power of autosomal STRs is low. Therefore, X-STRs serve as an additional set of markers to increase the likelihood ratio (LR) (Alwi et al., 2023) since fathers and sons do not inherit X-chromosomal alleles from a common ancestor (Gomes *et al*., 2012).

The limited availability of commercial X-STR markers, in contrast to autosomal markers, imposes constraints on the discriminatory capacity of X-STR analysis. The most popular kit for X‐STR analysis is the Investigator Argus X‐12 Kit (Hakim et al., 2021) which integrates three markers from the GHEP-ISFG decaplex (Gusmão et al., 2008) along with nine additional X‐STRs. Other multiplexes include the Microreader™ 19X ID System kit (Lin et al., 2020), AGCU X19 STR kit (Zhang et al., 2016; Li *et al*., 2017), and Goldeneye 17X kit (Gao et al., 2019), all of which have been extensively studied in the Chinese population. However, a common challenge with these kits is that the markers are in linked groups (LG), requiring researchers to calculate recombination rates for specific populations. This is necessary to accurately calculate the LR, which is crucial for interpreting the results. Most commercial kits use X-STRs from four main linkage groups located at Xp22, Xq12, Xq26, and Xq28. However, these X-STRs may not provide sufficient information when mutations or recombination events occur within a linkage group (Yang et al., 2021), even though they can still be useful in very specific cases. Also, the large size of some amplicons may hinder the use of these kits if DNA is highly degraded. Therefore, expanding the repertoire of X-STR markers and improving the efficiency of multiplexing strategies contribute to a better resolution in forensic investigations (Szibor et al., 2003; Pinto et al., 2011; Yang *et al*., 2017).

Despite the existence of 55 described X-STR markers (as documented at http://www.chrx-str.org), only 12 have undergone extensive study (Crnjac et al., 2016; Hakim et al., 2021). Our work seeks to expand the number of markers by evaluating new X-STR markers in a sample of an admixed population from Rio de Janeiro, Brazil, using capillary electrophoresis and Massively Parallel Sequencing (MPS).

MPS is a rapidly advancing technique with significant potential in forensic applications (Bruijns et al., 2018). One of its key advantages is the ability to analyze sequences alongside repetition numbers, allowing for the distinction of isoalleles. MPS also enables amplification of smaller amplicon sizes, crucial for working with degraded samples, as seen in forensic scenarios involving exhumed bodies or victims of mass disasters. Additionally, MPS aids in identifying contributors in mixed DNA samples, improving resolution in complex forensic cases. Using this technology we identified 12 new X-STR markers that meet the criteria to complement the autosomal STR analysis and two loci characterized by high polymorphism, with numerous isoalleles distinguishable by their sequences. 

## Material and Methods

### X-STR markers

We evaluated the forensic efficiency of a new 12 nonlinked X-STRs multiplex system, based on a genetic map using the range of recombination rates on X-Chromosome provided by Medina-Acosta (Machado and Medina-Acosta 2009). These X-STR markers have the potential to improve the efficiency of multiplexing strategies and contribute to a better resolution in forensic investigations. Primers were designed and selected according to the annealing temperature and amplicon size, to be able to analyze all markers in a single PCR reaction and run the amplicons in a single capillary electrophoresis (Table S1).

### Primer design

Primers for the target sequences were designed using OligoPerfect™ Designer from Invitrogen™ (http://www.invitrogen.com/). The list of primers used for CE and MPS sequencing and their characteristics are presented in Tables S1 and S2.

### Study population

Blood samples on FTA^®^ paper were obtained from 104 unrelated women and 100 unrelated men from Rio de Janeiro. Voluntary donation followed the guidelines set by the research ethics committee. The study was approved by the Research Ethics Committee of the UFRJ (Process No. 214/03).

### DNA extraction and quantification

The extraction method varied depending on the sample type. DNA was extracted from blood samples on FTA^®^ paper using the QIAamp DNA Investigator Kit (QIAGEN). Total DNA concentration was determined using NanoDrop^®^ spectrophotometry, and samples were stored at -20 ºC.

### Amplification and fragment analysis

The reactions were standardized according to the physical-chemical characteristics of the primers (Tables S1 and S2). The reactions were carried out using the Qiagen PCR Multiplex Mastermix kit, following the manufacturer’s instructions. For a reaction volume of 10 µL, 100 pmol of each primer (forward and reverse), and 10 ng of genomic DNA were added, with the following cycle: initial denaturation at 95ºC for 10 min was followed by 10 cycles of 94 °C for 30 s, 56 °C for 45 s, and 72 °C for 45 s, 25 cycles of 94 °C for 30 s, 60 °C for 45 s, and 72 °C for 60 s and a final extension at 72 °C for 20 min. Each amplification was performed separately for MPS analysis to avoid any preferential amplification of a particular sample, and then an amplicon pool was created for each locus. 

Capillary electrophoresis was used for fragment analysis using 10 ng of extracted DNA on the ABI 3500 Genetic Analyzer (Applied Biosystems) platform using four fluorophores, blue (6FAM), green (VIC), yellow (NED), or red (PET). Electropherograms were analyzed with GeneMapper ID-X v1.4. 

### Allelic frequencies, population and forensic parameters

Allelic frequencies and population parameters were determined using Genetic Data Analysis - GDA v1.1 (https://mybiosoftware.com/gda-1-1-genetic-data-analysis.html). Significance value (p) and linkage disequilibrium (LD) were recalculated with Bonferroni correction based on the number of independent tests (Weir 1996) conducted in each analysis, resulting in the corrected value of α’ = 0.0042. Forensic parameters were calculated using the online calculator available at http://www.chrx-str.org/.

### Allelic sequencing

Sequencing was performed on a subset of 94 individuals (27 males and 67 females) selected from a larger pool previously X-STR genotyped by CE. For each marker, at least two representatives per allele were chosen based on the allelic profiles obtained from CE data. This subset was selected to capture the full allelic diversity observed in the population. By targeting the minimal number of samples necessary to represent all detected alleles, this strategy ensured comprehensive genetic representation while optimizing cost-efficiency.

### MPS sequencing and analysis

The amplification fragments were sequenced in multiplex using the MiSeq platform (Illumina) at the Center of Human Identification Laboratory, UNTHSC (Texas). Following amplification product purification, libraries were prepared utilizing the TruSeq DNA PCR-Free High Throughput Library Prep Kit (Illumina®). The libraries were sequenced using the MiSeq Reagent Kit v3 (Illumina®), following the manufacturer’s protocol, on the MiSeq System platform (Illumina®). Sequencing data was analyzed using STRaitRazor software (King et al., 2017), for the characterization of sequence polymorphisms analysis in repetitive and flanking regions.

## Results 

### X-STRs loci characterization

**Figure 1 -:**
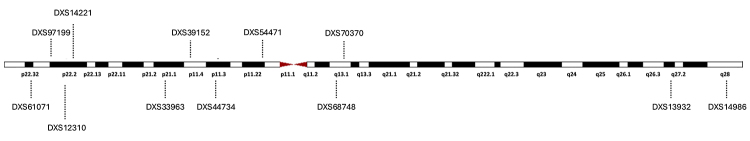
Ideogram showing the 12 X-STR markers and their loci across the X-chromosome.

The 12 markers are depicted in Figure 1 and Table 1 summarizes the information on these markers. The distance between neighbor markers ranged from 1.1 to 20.6 cM, with the shorter distance being between DXS68748 and DXS70370, apart by about 1.1 cM. Our analysis indicates that these two markers are not in LD.

Eight markers are located on the short arm of the X-chromosome and four on the long arm. Seven of the 12 markers are composed of tetranucleotide, and five markers are pentanucleotide repeats, one of which is a complex STR (DXS12310).

**Table 1 -:** Properties of the new proposed X-STR markers.

Marker	Distance (cM)	Motif	Position of the Repeat Core (hg38)		Reference Allele (hg38)
			Start	End	
DXS61071		(AAGG)n	6179100	6179127	7
	3.6				
DXS97199		(AATAG)n	9791972	9792156	37
	2.6				
DXS12310		(CATAG)nAG(CATAG)n	12382476	12382577	20
	1.9				
DXS14221		(TTTC)n	14293692	14293747	14
	19.7				
DXS33963		(TCTA)n	34035665	34035700	11
	5.4				
DXS39152		(TTCTA)n	39408811	39408811	16
	5.6				
DXS44734		(TAAA)n	44990555	44990590	9
	9.4				
DXS54471		(AAAT)n	54428216	54428259	11
	centromere				
DXS68748		(TTTTA)n	69612262	69612441	16
	1.6				
DXS70370		(TTTC)n	71234361	71234424	26
	69.2				
DXS13932		(TTTTA)n	140416505	140416571	13
	10.5				
DXS14986		(TTTC)n	150949067	150949166	24

Currently, X-STR data are available for many human populations (Gomes et al., 2020), and most of the data were obtained using the commercially available kit, the Investigator Argus X-12 kit (Qiagen GmbH, Germany). Presently, 55 markers are available (www.chrx-str.org, accessed in January 2025), with the majority located on the long arm (32 markers), 6 in the centromeric region, and 22 on the short arm. Most of these markers are tri- or tetranucleotide repeat sequences.

The markers we identified are primarily on the short arm and complement the existing set, which works with markers mostly on the long arm of the X-chromosome. Additionally, all our markers feature tetra- and pentanucleotide repeat sequences, which are less prone to artifacts such as stutter during analysis. This quality is particularly advantageous in forensic mixed DNA samples (Puente et al., 2017).

### Population data and forensic parameters

We conducted population studies with samples from 204 unrelated individuals from Rio de Janeiro, which included 104 females and 100 males. Forensic efficiency and population parameters based on allele frequencies are shown in Table 2.

The genotyping process of the 12 markers yielded a total of 106 alleles, with their respective frequencies shown in Table 2. Among these markers, those with more alleles were DXS64879 and DXS12310, each with 15 alleles, while the one with fewer alleles was DXS33963, with 7 alleles.

**Table 2 -:** Population genetic data of forensic efficiency parameters for the proposed 12 X-STR markers in a sample population of Rio de Janeiro.

Allele	DXS61071	DXS97199	DXS12310	DXS14221	DXS33963	DXS39152	DXS44734	DXS54471	DXS68748	DXS70370	DXS13932	DXS14986	Allele
7	0.167				0.052		0.051	0.007			0.143		7
8					0.069		????	0.042			0.047		8
9				0.013	0.322		0.021	0.087	0.014		0.547		9
10	0.007			0.010	0.189		0.188	0.350	0.024	0.047	0.183		10
11	0.010			0.007	0.320		0.329	0.347	0.155	0.104	0.053		11
11.2												0.020	11.2
12	0.041			0.050	0.048		0.312	0.160	0.185	0.108	0.027	0.017	12
12.2												0.007	12.2
13	0.048			0.040		0.040	0.082	0.007	0.149	0.152		0.027	13
14	0.137	0.077		0.097		0.166	0.017		0.172	0.185		0.051	14
14.2												0.132	14.2
15	0.198	0.040		0.182		0.266			0.203	0.138		0.034	15
15.2												0.017	15.2
16	0.120	0.114		0.357		0.226			0.081	0.135		0.169	16
16.2												0.081	16.2
17	0.217	0.270	0.041	0.167		0.179			0.017	0.091		0.148	17
18	0.041	0.237	0.041	0.077		0.083				0.040		0.195	18
19	0.014	0.144	0.058			0.040						0.061	19
20		0.064	0.192									0.017	20
20.2												0.003	20.2
21		0.054	0.236										21
22			0.209										22
23			0.182										23
24			0.017									0.014	24
25			0.024									0.007	25
Allele	DXS61071	DXS97199	DXS12310	DXS14221	DXS33963	DXS39152	DXS44734	DXS54471	DXS68748	DXS70370	DXS13932	DXS14986	Allele
N	292	299	292	300	291	301	292	289	296	297	300	296	N
PIC	0.829	0.800	0.800	0.767	0.707	0.781	0.709	0.675	0.820	0.857	0.603	0.866	PIC
h	0.153	0.178	0.177	0.208	0.252	0.192	0.251	0.278	0.159	0.129	0.359	0.122	h
HET	0.847	0.822	0.823	0.792	0.748	0.808	0.749	0.722	0.841	0.871	0.641	0.878	HET
PE	0.689	0.641	0.643	0.584	0.507	0.615	0.508	0.464	0.677	0.737	0.343	0.750	PE
PI	0.077	0.089	0.088	0.104	0.126	0.096	0.125	0.139	0.080	0.065	0.179	0.061	PI
aPDF	0.958	0.946	0.945	0.931	0.896	0.936	0.897	0.876	0.954	0.969	0.833	0.973	aPDF
bPDM	0.847	0.822	0.823	0.792	0.748	0.808	0.749	0.722	0.841	0.871	0.641	0.878	bPDM
MECT	0.829	0.800	0.800	0.767	0.707	0.781	0.709	0.675	0.820	0.857	0.603	0.866	MECT
MECD	0.721	0.683	0.682	0.641	0.570	0.658	0.572	0.534	0.709	0.760	0.455	0.774	MECD
*P* (HWE)	0.7558	0.0000	0.2229	0.0075	0.0294	0.4653	0.0009	0.0651	0.0168	0.0221	0.0021	0.0001	*P* (HWE)

N: Number of Chromosomes. PIC: Polymorphism Information Content. h: Homozygozity. Het: Heterozygosity. PE: Power of Exclusion. PI: Paternity Index. PDF: Power of Discrimination Female. PDM: Power of Discrimination Male. MECT: Mean Exclusion Chance in trios involving daughters. MECD: Mean Exclusion Chance in father/daughter duos. *P*(HWE): p value for Hardy-Weinberg Equilibrium exact test (P-value < 0.0042 in bold). ^a^ Only female’s data is used for the analysis. bOnly male data is used for the analysis.

The genotype frequency distributions exhibited no significant deviation from the Hardy-Weinberg equilibrium for most of the markers examined (p < 0.0042 after Bonferroni correction), except for DXS97199, DXS44734, DXS13932, and DXS14986.

The polymorphism information content (PIC), mean excluding chance (MEC), and observed heterozygosity (HET) for each STR are also shown in Table 2, along with the power of discrimination (PD) for females (PD_F_) and males (PD_M_), for each STR.

The HET ranged from 0.64 to 0.97, being DXS70370 the marker with higher heterozygosity. The PD values ranged from 0.94 (DXS39152) to 0.98 (DXS61071) in females and 0.76 (DXS33963) to 0.89 (DXS64979) in males (Table 2). The PE ranged from 0.61 (DXS39152) to 0.76 (DXS64879). The forensic efficiency evaluation for our multiplex panel revealed high values of the combined power of discrimination in males (cPDM ≥ 0.999999996959049) and females (cPDF ≥ 0.999999999999995), as well as combined paternity exclusion (cPE ≥ 0.99999). Additionally, the mean exclusion chance in trios (MECT ≥ 0.99999999) and duos (MECD ≥ 0.999997) were also calculated.

Closely located markers are in linkage when they are more likely to be inherited together as a unit rather than independently. This linkage often results in a higher likelihood of being in LD. Both linkage and LD must be considered when interpreting relationships based on X-STR markers (Tillmar et al., 2008).

Since X-chromosome markers behave as autosomal markers in females and lineage markers in males, additional effort is needed to examine haplotype frequencies and the extent of LD. The commercially available Argus X-12 kit (Qiagen, Hilden, Germany) comprises 12 X-STRs organized into four linkage groups. Most studies suggest that each set of markers in this kit should be genotyped as a haplotype (Edelmann et al., 2012) whereas some population studies have shown the occurrence of LD between markers within the same linkage group (Tomas et al., 2012; Červenák et al., 2023). These findings indicate that the most used markers can be less informative than non-linked ones. The latter is supposed to be more informative (Garcia et al., 2022). Most studies done in the Brazilian population use a decaplex system previously described by Gusmao et al. (2009) which comprises only 6 markers on the short arm. Currently, the Argus X-12 kit is the one most used.

Our markers, including the four that deviated from HWE, are not included in linkage groups and do not exhibit LD, as they are separated by a genetic distance of at least 1.1 cM, which is long enough to consider that they all segregate independently during cell division, which adds value to any genotyping system (Martins et al., 2008; Rodrigues et al., 2008; Gusmão et al., 2009; Martins *et al*., 2009; Cainé et al., 2010; Silva et al., 2010; Penna et al., 2012; Martins *et al*., 2017; Garcia et al., 2024). 

### Allelic sequencing through MPS

The adoption of MPS provides a new perspective by revealing sequence variation in both the STR and its flanking regions, which increases the technique’s discriminatory strength. For allele characterization and to assure allele designation, we employed MPS methodology to build an allelic ladder displaying all the different alleles detected for each analyzed marker.

To this end, we sequenced 161 X-chromosomes, identifying 121 distinct alleles (Table S3) to facilitate the precise identification of intermediate alleles. Notably, certain alleles - specifically alleles 8 and 9 of marker DXS61071, allele 8 of marker DXS44734, and alleles 6, 7, and 15 of marker DXS54471 - were absent from our sample population and therefore they are not represented in the allelic sequence description. Through sequencing efforts, we identified 6 isoalleles of marker DXS14986 and 9 of marker DXS12310 (as outlined in Table S3) by analyzing the repeat structure.

Additionally, we detected SNPs within the repeat and flanking regions of markers DXS61071, DXS97199, DXS33963, and DXS14986. We next compared these findings with data from the 1000 Genomes Project database (accessible at https://www.internationalgenome.org). Table 3 displays the SNPs associated with each marker, including details such as the reference and alternative alleles and the population frequency of the observed variations according to the database.

**Table 3 -:** SNPs in the repeat or flanking region of X-STR markers and their frequency in 1000 genomes populations.

STR locus	SNP location	SNP ID	1000 Genomes variant data							
			Reference allele	Alternative allele	Alternative allele frequency	AMR	EUR	SAS	EAS	AFR
DXS61071	Flanking	rs5916322*	A	G	0.65	0.71	0.63	0.54	0.45	0.86
DXS97199	Repeat	rs377423633	T	A	0.62	0.66	0.79	0.47	0.52	0.67
		rs371141960*	T	A	0.66	0.69	0.80	0.48	0.53	0.74
	Flanking	rs113651860	T	A	0.57	0.58	0.64	0.38	0.45	0.72
DXS33963	Flanking	rs3124163*	A	C	0.54	0.49	0.71	0.46	0.47	0.56
DXS14986	Repeat	rs200424800	T	C	0.33	0.34	0.44	0.39	0.40	0.15
	Flanking	rs202004750*	T	C	0.36	0.36	0.44	0.39	0.40	0.24
		rs1202686*	C	T	0.14	0.09	0.03	0.04	0.05	0.38

*SNPs detected in both our samples and in the 1000 Genomes population sample.

AMR: American population; EUR: European population; EAS: East Asian population; AFR: African population.

We identified five SNPs associated with the STR markers in our sample that had been previously documented. The database reports three additional common SNPs (with a frequency over 33%) that could potentially be analyzed using our multiplex assay. However, these SNPs were not found in our population sample. We focused solely on SNPs with a higher frequency (>10%), as rare alleles typically do not significantly contribute to allelic diversity (Moura-Neto et al., 2021).

## Discussion

Studies using commercial autosomal STR kits in different populations already pointed to the increase in the number of alleles when analyzing STR using MPS technology (Novroski et al., 2016; Devesse et al., 2018; Gettings et al., 2018; Phillips et al., 2018; Silva et al., 2018; Barrio et al., 2019; Kwon et al., 2021; Moura-Neto et al., 2021).

It has been shown that this technique provides new information since it allows us to investigate the number of repeats and the structure of the repeat. Additionally, it is possible to analyze the SNPs within the repeat and in the flanking region of a STR marker.

Our findings align with previous research indicating that MPS sequencing offers deeper insights into STR markers, both in terms of length and sequence information. Our study suggests that incorporating these new markers in conjunction with MPS analysis could potentially increase the power of discrimination in forensic analysis. This is attributed to the capability of the sequencing technique to differentiate isoalleles and to facilitate the deconvolution of mixed samples.

This study aimed to expand the array of X-STR markers available for forensic analysis. We introduced 12 novel X-STR loci that meet the criteria to complement autosomal STR analysis. Among these, two loci demonstrated high polymorphism with multiple isoalleles distinguishable by sequence, detectable throughout MPS technology. Our study evaluates these markers in an admixed population from Rio de Janeiro, Brazil - a population with a complex genetic structure resulting from European, African, and Native American contributions. This is particularly relevant because most existing data on these markers come from studies in the Chinese population, which has a distinct and more homogeneous genetic background. As a result, allele frequency distributions and polymorphism patterns may differ substantially, influencing the reliability and informativeness of these loci for forensic use in diverse populations. Our findings provide crucial population-specific data to support the broader applicability of these X-STR markers. No deviations from the Hardy-Weinberg equilibrium were observed except for the DXS101 locus. Due to the very high number of alleles at DXS101, the relatively small population size might have been inadequate to produce reliable allele frequencies (Martins et al., 2008). Such deviations are not unexpected in highly polymorphic loci analyzed in limited samples, as the statistical power of HWE tests decreases with increasing allelic diversity (Wigginton et al.,, 2005). In admixed populations, like that of Rio de Janeiro, deviations may also reflect subtle effects of population structure, further complicating equilibrium assessments. The genotype frequency distributions for female data showed no significant deviation from the Hardy-Weinberg equilibrium for most markers investigated (p > 0.05), except for DXS7130 (p = 0.005) and DXS10011 (p = 0.001). Exact tests of population differentiation between allele frequencies demonstrated that the allele frequencies observed between males did not present significant differences from the frequencies observed for females for any of the markers investigated (Rodrigues et al., 2008). These findings may be due to population admixture or substructure (Deng et al., 2001).

The findings of this study must be interpreted in the context of the limitations of our sample. For the population studies, we tested 200 individuals (100 males and 104 females) from an admixed population, specifically the population of Rio de Janeiro. This sample size limitation may have contributed to the deviation from Hardy-Weinberg Equilibrium (HWE) observed in four of the markers described in this study. Despite our relatively small sample size of 161 X-chromosomes, through MPS we identified SNPs within the repeat and flanking regions in four of the markers while also revealing distinct patterns of repeat structure within two markers. It is conceivable that with a larger sample size, we could uncover additional SNPs associated with our population, thereby potentially increasing the PD of the X-STR markers.

In conclusion, we developed and evaluated the forensic efficiency of a new X-STRs multiplex system, that may increase the discrimination power for mixtures and complex cases. Moving forward, further investigation should explore the usefulness of these markers in other populations.

## Supplementary material

The following online material is available for this article:

Table S1 - Information of the 24 primers used for CE.

Table S2 - Information of the 24 primer pairs used for NGS.

Table S3 - String sequence information for all characterized alleles through MPS.

## Data Availability

 All FastQ files are available under request, for collaboration purposes.
