# Supplementary material for: Characterization of a novel unliked 12 X-STR typing assay for forensic purposes in an admixed Rio de Janeiro population sample
Source: Genet Mol Biol. 2025 Dec 12;48(4):e20250015. doi: 10.1590/1678-4685-GMB-2025-0015 (PMC12704242; doi:10.1590/1678-4685-GMB-2025-0015)
Supplement: Table S1 - [file 1415-4757-GMB-48-04-e20250015-s1.pdf]

Genetics and Molecular Biology
https://doi.org/10.1590/1678-4685-GMB-2025-0015

Supplementary Material to "Characterization of a novel unliked 12 X-STR typing assay for forensic purposes in an admixed Rio de Janeiro population sample"

Table S1 - Information of the 24 primer pairs used for CE.

STR locus	Dye label		Primer sequence	T_m	Repeat unit
DXS97360	NED	F:	CCTGTCAATACTGTGCTTTGC	52.4	(TTTA)12
		R:	GCCTGGTAACAGAGCGAGAC	55.9	
DXS11062	PET	F:	TCAAGACCAGCCTAGGCAAT	51.8	(GGAA)5(GAAA)21(GAGA)1
		R:	GCGGTTCTGAATCTTGGCTGT	54.4	
DXS13932	6FAM	F:	ACTGGGCCTGATCTTTTTCA	49.7	(TTTTA)14
		R:	GTCTGAACAAGGGAGACAGCA	54.4	
DXS14221	NED	F:	TGCATGCAGAATCCATTTGA	47.7	(TTTC)18
		R:	GCCTGGGCAACAAGAGTGAA	53.8	
DXS33963	6FAM	F:	GCCTGGGTCAAAAGTTATAGGA	53.0	(TCTA)15
		R:	GTGATTGAAATTCTCTGTTGTAAGTGA	53.7	
DXS54471	6FAM	F:	GCCTGGAAACATAGCAAGACTC	54.8	(TAAA)9
		R:	GACGTGGCCCTGTGATAACTT	54.4	
DXS11846	VIC	F:	TCTGTGTTTGCTTGTCACTGC	52.4	(TTTA)9
		R:	GCCAGCTACTCAGGAGGGAGT	58.3	
DXS13664	NED	F:	CCTGGACATTTGTATGGCTTT	50.5	(TTTC)n
		R:	GCAAGGAACCCAAACAACTCAA	53.0	
DXS14986	PET	F:	GTTTGGCAAGTGACACGAGA	51.8	(TTTC)17
		R:	GCTGAGGCTAGGAGCTTGAGG	58.3	
DXS97199	6FAM	F:	GGTTGTGGTGAGCTGGAGAT	53.8	(AATAG)69
		R:	GCCAGAATACACCCTCCCTGT	56.3	
DXS85322	NED	F:	CCCAAACATAGATGGTGCATT	50.5	(TCTA)11
		R:	GGGAGGCTAGGGGAGAGATA	55.9	
DXS49546	6FAM	F:	TGCCATTTTGCATTCTGAAG	47.7	(CTTT)4(CT)3(CTTT)10
		R:	GAAGGGAGGAAAGCAGGAAAG	54.4	
DXS64879	VIC	F:	CTGGACCTGGCACTCTCACT	55.9	(TTCC)39
		R:	GAGGATTGCACCACTCCACTC	56.3	
DXS87655	NED	F:	TTGATTCCTCACAGAAGTCAGC	53	(TAGA)14
		R:	GCATTAGCATGTGGGGCAATA	52.4	
DXS61071	6FAM	F:	CCTGAGCGACAAAGCAAGAC	53.8	(GGAA)8
		R:	GCAAAATGAGTCGATGGGTGTT	53	
DXS12310	VIC	F:	GGATTGCATACTTGGGGATG	51.8	(CATAG)23
		R:	GTAAGCAACACCCAGCACAGA	54.4	
DXS27425	PET	F:	CCCTTGGCAAACACCATTAT	49.7	(CTTT)4(CCTT)7(CTTT)7
		R:	GGCAGAAGAATCGCTTGAAC	51.8	

STR locus	Dye label		Primer sequence	T_m	Repeat unit
DXS28157	6FAM	F:	GCCCAGTGAGGATGCATTA	51.1	(TAAA)9
		R:	GTTTGTGCTTATTATTTCTTCCTGAGA	53.7	
DXS35197	NED	F:	TCCTCCTTTTAGTGATTTATAGTGTCA	53.7	(TAGA)5(TGA)1(TAGA)1(TGA)1(TAGA)10
		R:	GTGGGACTTCTGAAACCCTGA	54.4	
DXS39152	PET	F:	CAGGAATCATGAAGCAGCAA	49.7	(CTATT)17
		R:	GTTCCTGGCCTTGATCAAATTA	51.1	
DXS40659	VIC	F:	TTCCCAGGAGTGAGTTTGCT	51.8	(TCTG)8(TCTA)18
		R:	GCAAGACCAGCCTGGGAAATA	54.4	
DXS44734	NED	F:	GCGCTTGTAATCCCAGCTAC	53.8	(TAAA)10
		R:	GCACGAGAACTTTGTGAGGA	51.8	
DXS62993	PET	F:	TGGGAAGAGGCAAAGAAAGA	49.7	(GAAA)21
		R:	GCGTCAAAGTGTGCATGTGTG	54.4	
DXS70370	VIC	F:	TGGAATTCACATCTGGTGGA	49.7	(TTTC)14
		R:	GCAGAGATCATGCCAATGCAC	54.4	
