# Supplementary material for: Characterization of a novel unliked 12 X-STR typing assay for forensic purposes in an admixed Rio de Janeiro population sample
Source: Genet Mol Biol. 2025 Dec 12;48(4):e20250015. doi: 10.1590/1678-4685-GMB-2025-0015 (PMC12704242; doi:10.1590/1678-4685-GMB-2025-0015)
Supplement: Table S2 - [file 1415-4757-GMB-48-04-e20250015-s2.pdf]

Genetics and Molecular Biology
https://doi.org/10.1590/1678-4685-GMB-2025-0015

Supplementary Material to "Characterization of a novel unliked 12 X-STR typing assay for forensic purposes in an admixed Rio de Janeiro population sample"

Table S2 - Information of the 24 primer pairs used for NGS.

STR locus	Sense	Primer sequence	T_m	Repeat unit	Amplicon size	Alellic range
DXS14221	F:	TGCATGCAGAATCCATTTGA	50	(TTTC)14	168-204	9-18
	R:	GCCTGGGCAACAAGAGTGAA	55			
DXS33963	F:	GCCTGGGTCAAAAGTTATAGGA	52	(TCTA)9 TGTA (TCTA)2	213-233	7-12
	R:	GTGATTGAAATTCTCTGTTGTAAGTGA	52			
DXS54471	F:	GCCTGGAAACATAGCAAGACTC	54	(TAAA)10	185-228	5-15.3
	R:	GACGTGGCCCTGTGATAACTT	54			
DXS68748	F:	GTGGGTATTCAGGGGTTGAC	52	(TTTTA)16	218-250	9-17
	R:	GGAAAATCGCTTGAACCAGA	51			
DXS14986	F:	GTTTGGCAAGTGACACGAGA	53	(TTTC)21	221-275	11.2-25
	R:	GCTGAGGCTAGGAGCTTGAGG	56			
DXS97199	F:	GGTTGTGGTGAGCTGGAGAT	54	(AATAG)37	172-207	14-21
	R:	GCCAGAATACACCCTCCCTGT	55			
DXS61071	F:	CCTGAGCGACAAAGCAAGAC	54	(GGAA)7	218-266	7-19
	R:	GCAAAATGAGTCGATGGGTGTT	54			
DXS12310	F:	GGATTGCATACTTGGGGATG	51	(CATAG)11 AG (CATAG)9	217-257	17-25
	R:	GTAAGCAACACCCAGCACAGA	55			
DXS39152	F:	CAGGAATCATGAAGCAGCAA	51	(CTATT)15	215-245	13-19
	R:	GTTCCTGGCCTTGATCAAATTA	51			
DXS44734	F:	GCGCTTGTAATCCCAGCTAC	53	(TAAA)9	226-254	7-14
	R:	GCACGAGAACTTTGTGAGGA	52			
DXS70370	F:	TGGAATTCACATCTGGTGGA	50	(TTTC)3 TCTT (TTTC)4 T (TTTC)16	158-190	10-18

STR locus	Sense	Primer sequence	T_m	Repeat unit	Amplicon size	Alellic range
	R:	GCAGAGATCATGCCAATGCAC	55			
DXS13932	F:	ACTGGGCCTGATCTTTTTCA	51	(TTTTA)10	185-210	7-12
	R:	GTCTGAACAAGGGAGACAGCA	54.4			
