# Supplementary material for: Characterization of a novel unliked 12 X-STR typing assay for forensic purposes in an admixed Rio de Janeiro population sample
Source: Genet Mol Biol. 2025 Dec 12;48(4):e20250015. doi: 10.1590/1678-4685-GMB-2025-0015 (PMC12704242; doi:10.1590/1678-4685-GMB-2025-0015)
Supplement: Table S3 - [file 1415-4757-GMB-48-04-e20250015-s3.pdf]

Genetics and Molecular Biology
https://doi.org/10.1590/1678-4685-GMB-2025-0015

Supplementary Material to "Characterization of a novel unliked 12 X-STR typing assay for forensic purposes in an admixed Rio de Janeiro population sample"

Table S3 - String sequence information for all characterized alleles through MPS.

Marker	Allele	Sequence
DXS14986	11.2	[TTTC]1 TC [TTTC]9 [TC]2
	12	[TTTC] [TTTTTC]1 [TTTC]1 TC [TTTC]8
	12.2	[TTTC]1 TC [TTTC]10 [TC]2
	13.2	[TTTC]1 TC [TTTC]11 [TC]2
	14	[TTTC]1 TC [TTTC]12 TC
	14.2'	[TTTC]1 TC [TTTC]12 [TC]2
	14.2	[TTTC]1 TC [TTTC]13
	15	[TTTC]1 TC [TTTC]13 [TC]1
	15.2'	[TTTC]1 TC [TTTC]13 [TC]2
	15.2	[TTTC]1 TC [TTTC]14
	16	[TTTC]1 TC [TTTC]14 [TC]
	16.2	[TTTC]1 TC [TTTC]15
	17	[TTTC]1 [TTTTTC]1 [TTTC]1 TC [TTTC]13
	17'	[TTTC]1 TC [TTTC]15 [TC]1
	18'	[TTTC]1 TC [TTTC]16 [TC]1
	18	[TTTC] [TTTTTC]1 [TTTC]1 TC [TTTC]14
	19'	[TTTC]1 TC [TTTC]17 [TC]1
	19	[TTTC] [TTTTTC]1 [TTTC]1 TC [TTTC]15
	20	[TTTC] [TTTTTC]1 [TTTC]1 TC [TTTC]4 [TT T/C C] [TTTC]11
	20'	[TTTC]1 TC [TTTC]18 [TC]1
	20.2	[TTTC]1 TC [TTTC]18 [TC]2
	24	[TTTC] [TTTTTC]1 [TTTC]1 TC [TTTC]4 [TT T/C C] [TTTC]15
	25	[TTTC] [TTTTTC]1 [TTTC]1 TC [TTTC]4 [TT T/C C] [TTTC]16
DXS 13932	7	[TTTTA]7
	8	[TTTTA]8
	9	[TTTTA]9
	10	[TTTTA]10
	11	[TTTTA]11
	12	[TTTTA]12
DXS14221	9	[CTTT]9
	10	[CTTT]10
	11	[CTTT]11
	12	[CTTT]12
	13	[CTTT]13
	14	[CTTT]14
	15	[CTTT]15
	16	[CTTT]16

Marker	Allele	Sequence
	17	[CTTT]17
	18	[CTTT]18
DXS33963	7	[TCTA]7
	8	[TCTA]8
	9	[TCTA]9
	10	[TCTA]10
	11	[TCTA]11
	12	[TCTA]12
DXS54471	7	[AAAT]7
	8	[AAAT]8
	9	[AAAT]9
	10	[AAAT]10
	11	[AAAT]11
	12	[AAAT]12
	13	[AAAT]13
DXS68748	9	[TTTTA]9
	10	[TTTTA]10
	11	[TTTTA]11
	12	[TTTTA]12
	13	[TTTTA]13
	14	[TTTTA]14
	15	[TTTTA]15
	16	[TTTTA]16
	17	[TTTTA]17
DXS97199	14	[AATAG]14
	15	[AATAG]15
	16	[AATAG]16
	17	[AATAG]17
	18	[AATAG]18
	19	[AATAG]19
	20	[AATAG]20
	21	[AATAG]21
DXS61071	7	[TTCC]7
	10	[TTCC]10
	11	[TTCC]11
	12	[TTCC]12
	13	[TTCC]13
	14	[TTCC]14
	15	[TTCC]15
	16	[TTCC]16
	17	[TTCC]17
	18	[TTCC]18
	19	[TTCC]19
DXS12310	17	[CATAG]6 AG [CATAG]11
	17'	[CATAG]5 AG [CATAG]12
	17''	[CATAG]11 AG [CATAG]6

Marker	Allele	Sequence
	18	[CATAG]11 AG [CATAG]7
	18'	[CATAG]6 AG [CATAG]12
	18"	[CATAG]7 AG [CATAG]11
	19	[CATAG]7 AG [CATAG]12
	20	[CATAG]7 AG [CATAG]13
	20'	[CATAG]10 AG [CATAG]10
	21	[CATAG]10 AG [CATAG]11
	21'	[CATAG]7 AG [CATAG]14
	22	[CATAG]11 AG [CATAG]11
	22'	[CATAG]12 AG [CATAG]10
	23	[CATAG]12 AG [CATAG]11
	23'	[CATAG]9 AG [CATAG]14
	24	[CATAG]10 AG [CATAG]14
	24'	[CATAG]9 AG [CATAG]13
	25	[CATAG]12 AG [CATAG]13
DXS39152	13	[TTCTA]13
	14	[TTCTA]14
	15	[TTCTA]15
	16	[TTCTA]16
	17	[TTCTA]17
	18	[TTCTA]18
	19	[TTCTA]19
DXS44734	7	[TAAA]7
	9	[TAAA]9
	10	[TAAA]10
	11	[TAAA]11
	12	[TAAA]12
	13	[TAAA]13
	14	[TAAA]14
DXS70370	10	[TTTC]10
	11	[TTTC]11
	12	[TTTC]12
	13	[TTTC]13
	14	[TTTC]14
	15	[TTTC]15
	16	[TTTC]16
	17	[TTTC]17
	18	[TTTC]18
